# Novel recombinant immunotoxin of EGFR specific nanobody fused with cucurmosin, construction and antitumor efficiency *in vitro*

**DOI:** 10.18632/oncotarget.16930

**Published:** 2017-04-07

**Authors:** Cuimin Deng, Jiani Xiong, Xiaofan Gu, Xiaoying Chen, Shuifa Wu, Zhe Wang, Duanduan Wang, Jinjin Tu, Jieming Xie

**Affiliations:** ^1^ Department of Pharmacology, Fujian Medical University, Fuzhou, Fujian, China; ^2^ Department of Oncology, Fujian Medical University Union Hospital, Fuzhou, Fujian, China; ^3^ Department of Experimental Teaching Center of Basic Medical Science, Fujian Medical University, Fuzhou, Fujian, China; ^4^ Department of Pharmacology, The 180th Hospital of PLA, Quanzhou, Fujian, China; ^5^ Department of Breast Surgery, Fujian Medical University Union Hospital, Fuzhou, Fujian, China

**Keywords:** immunotoxins, nanobody, EGFR, cucurmosin

## Abstract

Epidermal growth factor receptor (EGFR) overexpression is related to the increased aggressiveness, metastases, and poor prognosis in various cancers. In this study, we successfully constructed a new EGFR nanobody-based immunotoxin rE/CUS containing cucurmosin (CUS), The immunotoxin was expressed by prokaryotic system and we obtained a yield of 5 mg protein per liter expression medium. The percentage of it's binding ability totumor cell lines A549, HepG2, SW116, which highly expressed EGFR was 55.6%, 79.6% and 97.1%, respectively, but SW620 was only 4.45%. rE/CUS has the ability to bind A549, HepG2, SW116 cells specifically, and the antigen binding capability was not affected because of extra part of CUS component. The rE/CUS significantly inhibited the cell viability against EGFR over expression tumor cell lines in a dose-and time-dependent manner. Moreover, rE/CUS also induced apoptosis of HepG2 and A549 mightily. Our results demonstrate that rE/CUS is a potential therapeutic strategy for treating EGFR-positive solid tumors.

## INTRODUCTION

In 2012, approximately 14 million new cancer cases were recorded and almost 8.2 million people died from cancer all around the world. Malignant tumor has become one of the common causes of death in the US, and accounted for nearly 1 of every 4 deaths [1]. Those most fatal one are epithelial cells derived from lungs, liver, breasts, prostate, and stomach [2]. Although, treated with surgery, radiation, chemotherapy, and antibody-mediated receptor targeted therapy, etc., patients still suffer from unfavorable prognosis.

Epidermal growth factor receptor (EGFR, ErbB-1 or HER1) over -expression can be detected on numerous epithelium-originated tumor cells, especially those generated from respiratory and digestive systems [3]. It is clear that more than two third of breast cancers express EGFR abnormally [4], and 90% of pancreatic cancer patients, of which the 5-year survival rate is less than 5%, overly express EGFR or it's ligands, such as TGFα and EGF [5–6]. Non-small cell lung cancers (NSCLC) and head-and-neck cancers (HNC) also showed EGFR overexpression [7–8]. Glioblastomas exhibit EGFR overexpression in more than 4/5 of cases and more than half of those were tested with an additional expression of the EGFR deletion variant EGFRvIII [9–10]. Furthermore, as EGFR has been known to be pivotal for proliferation, cell survival, and vascularization, implicating that it is overexpression is a crucial factor for tumor initiation, progression and neovascularization [11], which means that the treatments aiming to blocking EGFR can be an effective way for curing cancer.

In the early 90 s, the heavy-chain antibodies (HCAbs, -95 kDa) were constructed by Hamers-Casterman, etc [12]. These synthetic antibodies consist of two identical heavy chains but without light chain. Unlike single-chain Fv(scFv), HCAbs, which retain high antigen-binding capacities, do not stick to each other or gather into pieces. The variable domain of the heavy chain from HcAbs (i.e.VHH), known as nanobodies or single domain antibodies, are well-functioned and they are considered to be the smallest nature-derived fragments able to combine the target protein to date [39]. Compared with conventional antibodies, the obvious advantages of nanobodies including low molecular weights, out-standing structural stability [13], and high solubility [14]. Moreover, it is easily available through phage display technology [15], and engineered into multivalent and multi-specific formats,and then can be fused with other proteins by recloning [16]. Based on the data collected by Ablynx NV (Belgium) in a Phase I trial, nanobodies have lower level of immunogenic potential for the reason that the amino acid sequence and the conformation of VHH and that of human VH of family III are extremely alike [17–18]. However small it is, nanobodies cannot penetrate cytomembranes freely. It is highly possible that the nanobodies target cancer cells through extracellular ligands or transmembrane proteins which were expressed abnormally compared with normal cells [39]. Nanobodies, such as those against EGFR [12], Her2 [19], VEGFR2 [20], c-Met [21], CXCR7 and MUC-1 [22], targeting transmembrane proteins and extracellular tumor-specific glycoproteins for cancer therapy, can be coupled with effector domain like toxins. In other words, it can be used as deliverys of conjugates for treating cancer or other diseases [23–26]. Therefore, nanobodies is in fact a targeting moiety of the whole parts of immunotoxins for cancer therapy.

Antibody-cytotoxic fusions or immunotoxins have been widely used in anti-tumor therapy nowadays [27], which mainly contains two compartments. One is the cytotoxin part which typically was plant-derived ribosomal inactivating protein (RIP) toxins (e.g. ricin, gelonin, and saporin), or bacterial toxins (e.g. diphtheria toxin, and *Pseudomonas* exotoxin A). The other part is usually consist of antibodies that against particular ligands [28]. This modified antibodies can function as receptors which have the ability to combine with it's ligands on the membrane surface, assisting toxin parts get into tumor cytoplasm. After that, toxin regions acquire their enzyme activities of protein synthesis inhibition, leading to tumor cell death [29–30].

Cucurmosin (CUS) extracted from pumpkin pulp is a basic alkaline glycoprotein with single polypeptide chain. After it's DNA sequences, amino acid, and the protein secondary and tertiary structures were analyzed [31–35], CUS was proved to be one of the type 1 ribosome inactivating proteins (RIPs) [36], lacking a galactose-binding lectin B subunit.

In this study, rE/CUS an immunotoxin, was generated by recombining the nanobody 7D12 and CUS. As a ligand competitive inhibitor, 7D12 epitope has the capability to take up the ligand-binding site on domain III of EGFR [37], blocking the combination of EGF to the EGFR and competing for the binding site of cetuximab [38]. In order to discuss the efficacy, rE/CUS was constructed and characterized to evaluate its potential antitumor activity.

## RESULTS

### Construction, expression, purification and identification of rE/CUS

**Figure 1:**
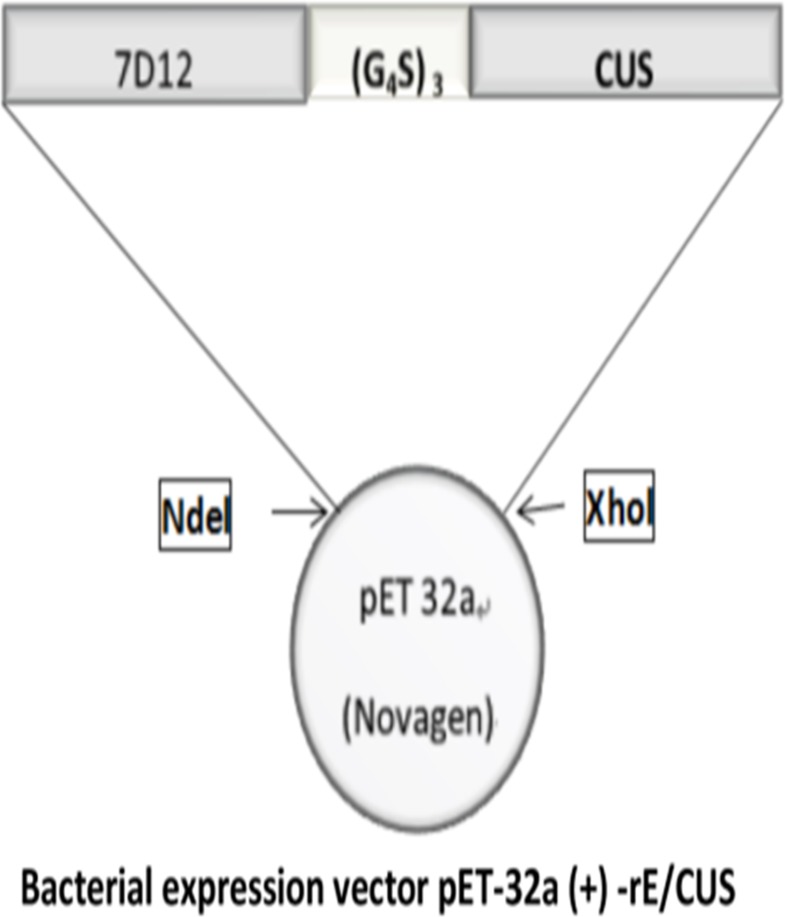
The schematic diagram of recombinant immunotoxin rE/CUS 7D12, EGFR specific nanobody. (G_4_S)_3_, flexible linkers consisting of glycine and serine residues; CUS, cucurmosin.

**Figure 2:**
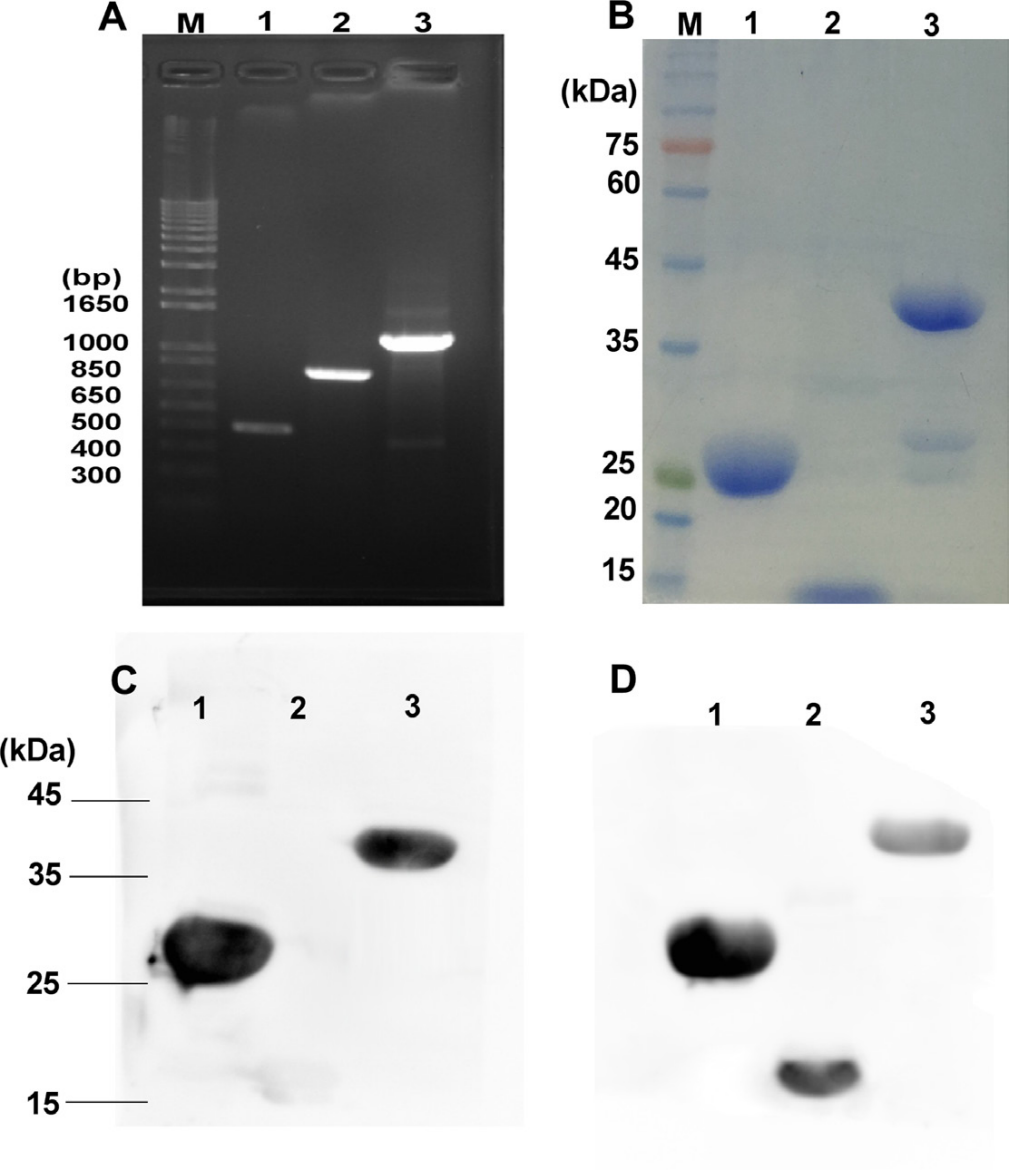
Construction, expression and purification of rE/CUS (**A**) Agarose gel for electrophoresis of all the amplified products, M: DNA maker, Lane 1: 7D12 PCR amplification products, lane 2: CUS PCR amplification products, lane 3: rE/CUS PCR amplification products. (**B**) SDS-PAGE analysis of the purification immunotoxin rE/CUS, M: protein maker, lane1: purification of CUS protein, lane 2: purification of 7D12 antibody, lane 3: purification of rE/CUS protein. (**C**) and (**D**) Western blot analysis identified the immunotoxin of rE/CUS, (C) lane 1: protein CUS, lane 2: protein 7D12, lane 3: protein rE/CUS, with mouse anti-CUS as the primary antibody, and goat anti-mouse HRP as the secondary antibody. (D) lane 1: protein CUS, lane 2: protein 7D12, lane 3: protein rE/CUS, with mouse anti-HIS as the primary antibody, and goat anti-mouse HRP as the secondary antibody.

rE/CUS is a chimeric protein composed of 7D12 fusing with a new type I RIP CUS, The -COOH terminal of 7D12 tethering the -NH2 terminal of CUS through a flexible linker (G_4_S)_3_ using overlapping PCR (Figure 1). The fusion protein was sequenced and verified by Vector NTI sequence alignment analysis. All the amplified products were transferred into agarose gel for electrophoresis (Figure 2A), then the fusion gene rE/CUS was cloned into pET-32a (+) and transfected into BL21 (DE3) E.coli cells and seduced by Isopropyl β-D-1-thiogalactopyranoside (IPTG). After the E.coli culture solution was collected, washed and eluted, the rE/CUS protein was then examined through sodium dodecyl sulfate polyacrylamide gel electrophoresis (SDS-PAGE). The expected molecular mass of 7D12, CUS and rE/CUS was about 15KDa, 25KDa and 42KDa respectively (Figure 2B). As shown in Figure 2, we successfully constructed a new recombinant immunotoxin rE/CUS, and yielded 5mg protein from 1L of bacterial culture, Ni+ affinity chromatography column was used for enriching target protein by trapping the 6×His tag on the terminal side of amino acids. CUS and rE/CUS was then migrated on 12% SDS-PAGE and identified through Western blot analysis (Figure 2C and 2D). The mouse anti-CUS and the mouse anti-HIS specific band appeared. This finding indicated the expressed protein was the expected immunotoxin.

### EGFR expression on cell lines

**Figure 3:**
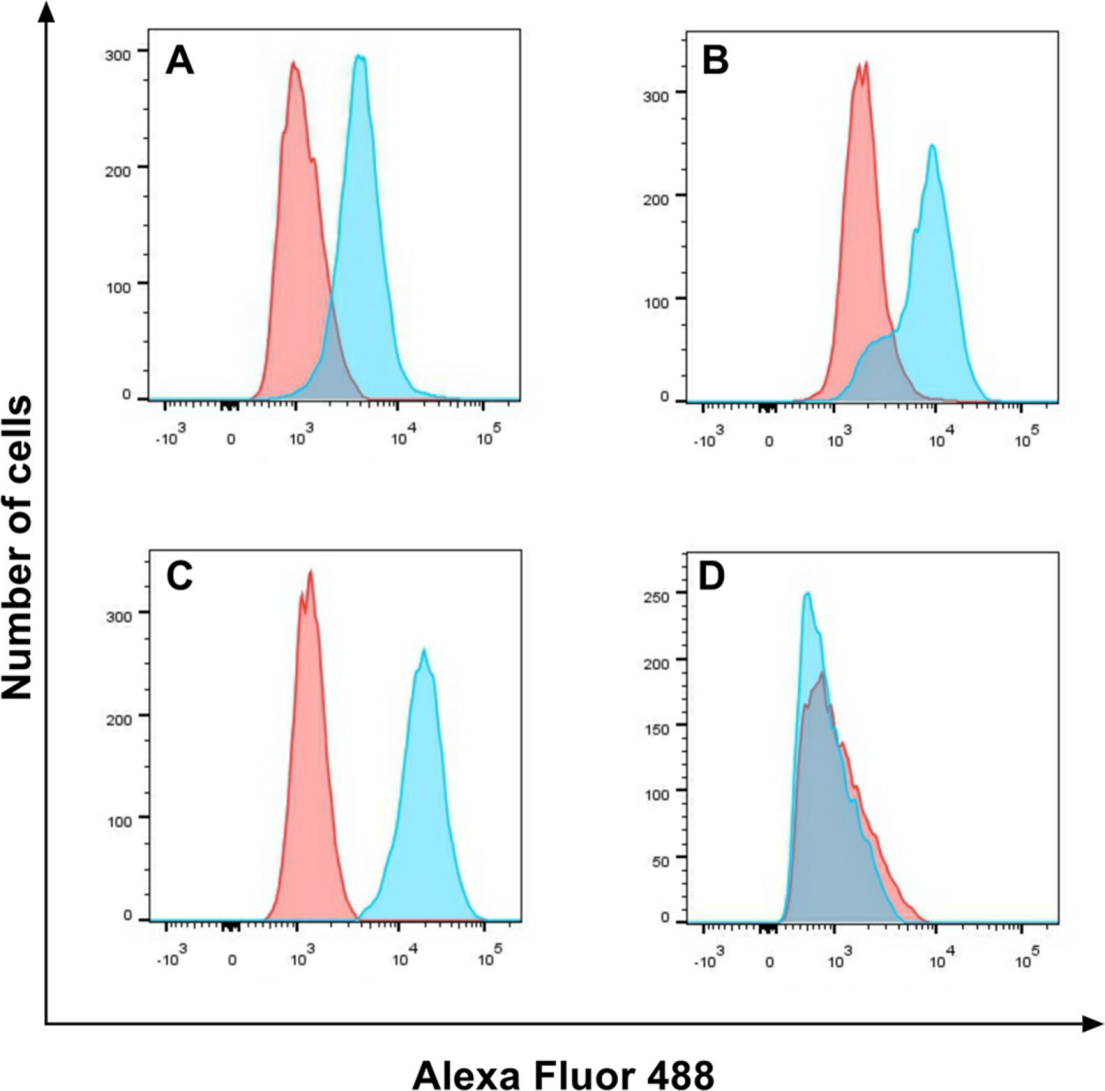
EGFR expression on cell lines EGFR expression on A549 (**A**), HepG2 (**B**), SW116 (**C**) and SW620 (**D**) was detected by flow cytometry, the red part was untreated group, and the blue part was treat group.

EGFR expression on cell lines (HepG2, A549, SW116 and SW620) was detected by Flow cytometry. The cetuximab was used as the primary antibody and the anti-human-FITC was used as the second antibody. The percentage of EGFR expression on tumor cell lines A549, HepG2, SW116 calculated from the corresponding dot plot was 55.6%, 79.6% and 97.1%, respectively, but SW620 was only 4.45% (Figure 3). As these data is shown, the positive cell lines A549, HepG2, SW116 was highly expression EGFR compared with negative cell line SW620.

### rE/CUS binding ability

**Figure 4:**
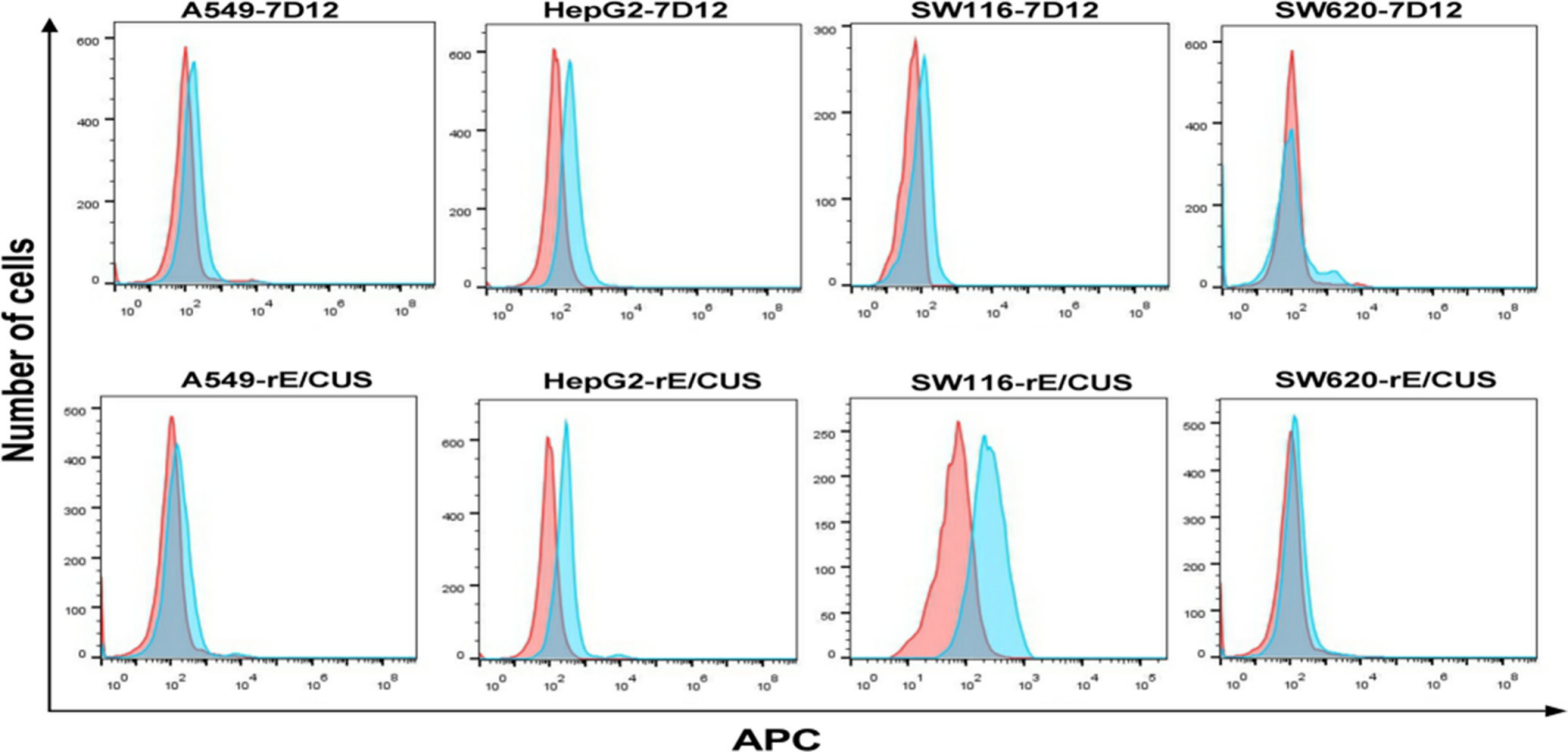
Analysis of binding ability of rE/CUS to tumor cell lines by Flow cytometry EGFR-positive cell lines HepG2, A549, SW116 and EGFR negative cell line SW620 were treated with 7D12 and rE/CUS. Cells were incubated with mouse anti-HIS antibody and the mouse anti-HIS-APC, then analyzed by Flow cytometry.

In order to prove that the reconstructed anti-EGFR antibody retained binding ability to EGFR after fusion, EGFR-positive cells lines HepG2, A549, SW116 and EGFR-negative cell line SW620 were treated with 30 μmol/L of 7D12 and rE/CUS, then mouse anti-HIS antibody and the goat anti-mouse-APC were added for reconstructed immunotoxin binding analyzing using Flow cytometry. Data displayed that 7D12 and rE/CUS were able to combine with EGFR positive cells HepG2, A549, SW116 but cannot conglutinate to negative cell line SW620 (Figure 4).

### *In vitro* cytotoxicity of rE/CUS

**Figure 5:**
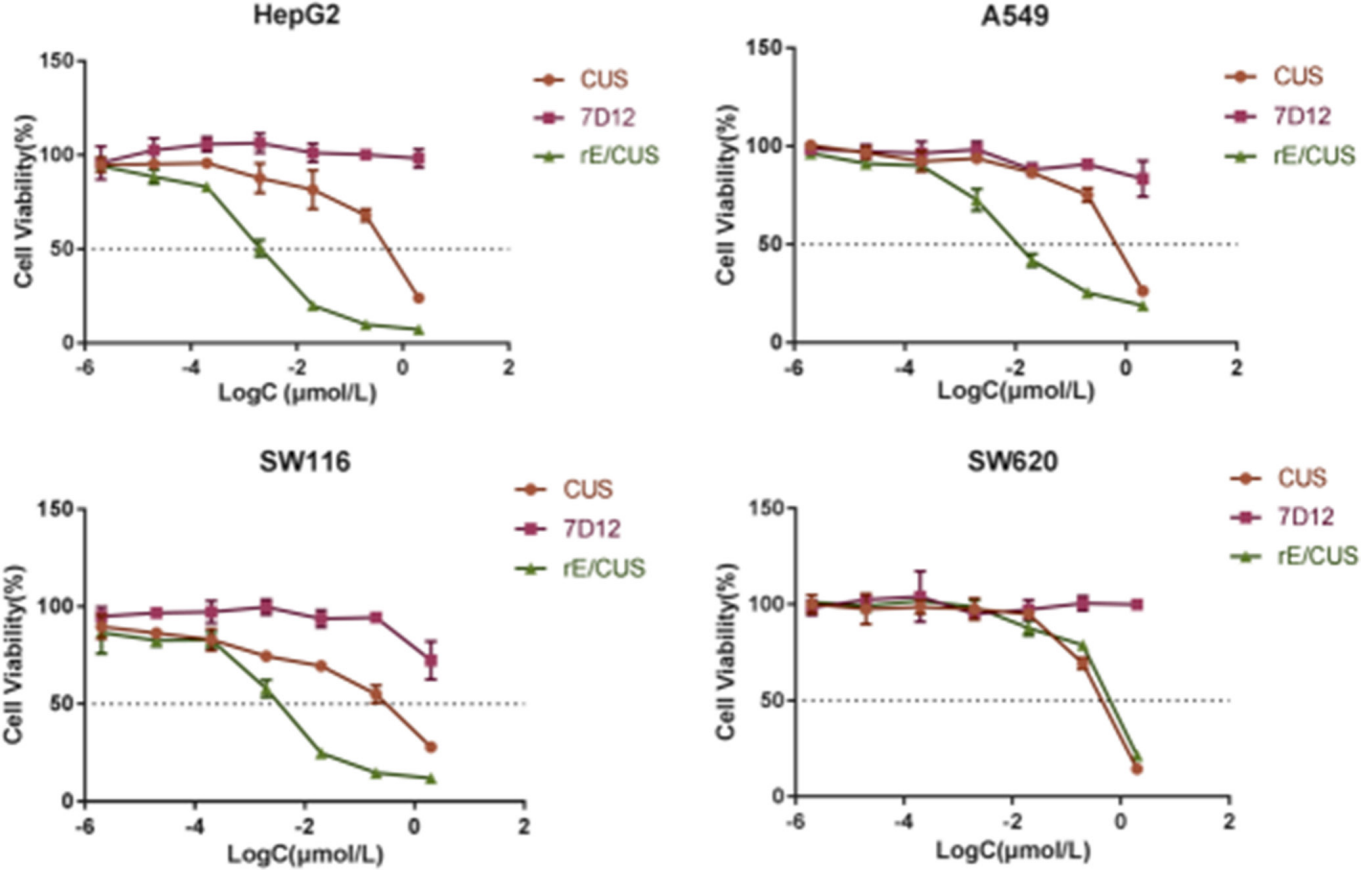
Cytotoxicity of rE/CUS in HepG2, A549, SW116 and SW620 cells through SRB assay log-normal distribution model is applied.The viability of cells is the percentage of cells alive after treating with rE/CUS, CUS and 7D12. Points represent the mean of 3 independent experiments; bars represent the SD.

Data shows that the immunotoxin rE/CUS remains antigen binding capability. We then evaluated the cytotoxicity of rE/CUS through SRB assay. The protein CUS, 7D12 and rE/CUS were tested by co-culturing with a number of tumor cell lines for 72 h (Figure 5). The results illustrates that CUS and rE/CUS can significantly inhibited the cells viability through a dose-dependent manner, but there is barely cytotoxin effects of 7D12 on the same cell lines. The IC50 of rE/CUS was significantly lower than CUS whose IC50 value act on HepG2, A549, SW116 was 217, 39, 89-fold higher than that of rE/CUS. The negative cell SW620 showed little cytotoxicity of rE/CUS compared with CUS itself (Table 1).

**Table 1:** SRB assay results of various tumor cell lines treated with gradient concentrations of CUS and rE/CUS for 72 h

cell line	IC50(nmol/L)		*P* value
	rE/CUS	CUS	
HepG2	2.15 ± 0.226	466.3 ± 0.483	0.0018^a^
A549	18.33 ± 0.018	716 ± 0.760	0.0006^a^
SW116	2.33 ± 0.032	208.33 ± 0.056	0.003^a^
SW620	463 ± 0.086	263 ± 0.044	0.0265

Data shown are IC50 (mean ± SD). ^a^ Compared with CUS, *p* < 0.05; SRB, sulforhodamine B.

**Figure 6:**
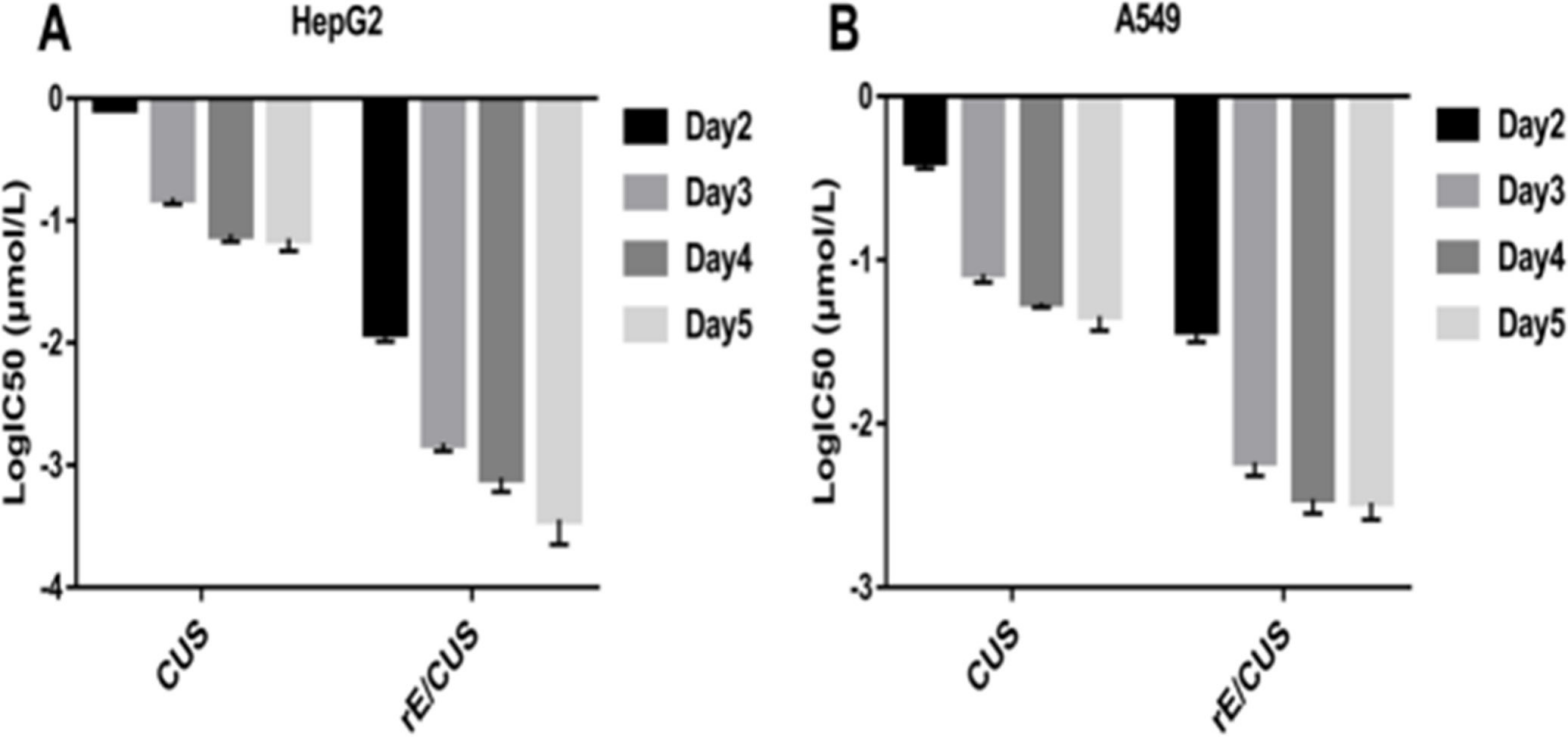
Dose-and time-dependent of the inhibitory effect of rE/CUS on the proliferation of HepG2 and A549 (**A** and **B**) IC50 values of CUS and rE/CUS in HepG2 and A549 for day 2-5 days.

The IC50 of rE/CUS, which reflects the it's cytotoxicity effects is not only dose dependent but time dependent (Figure 6), and were different with that of CUS in 2-5 days. Although both rE/CUS and CUS significantly reduced cell viability, rE/CUS elicited a greater effect on cell viability than CUS did.

### Cells inhibition of cell proliferation induced by rE/CUS via apoptosis

**Figure 7:**
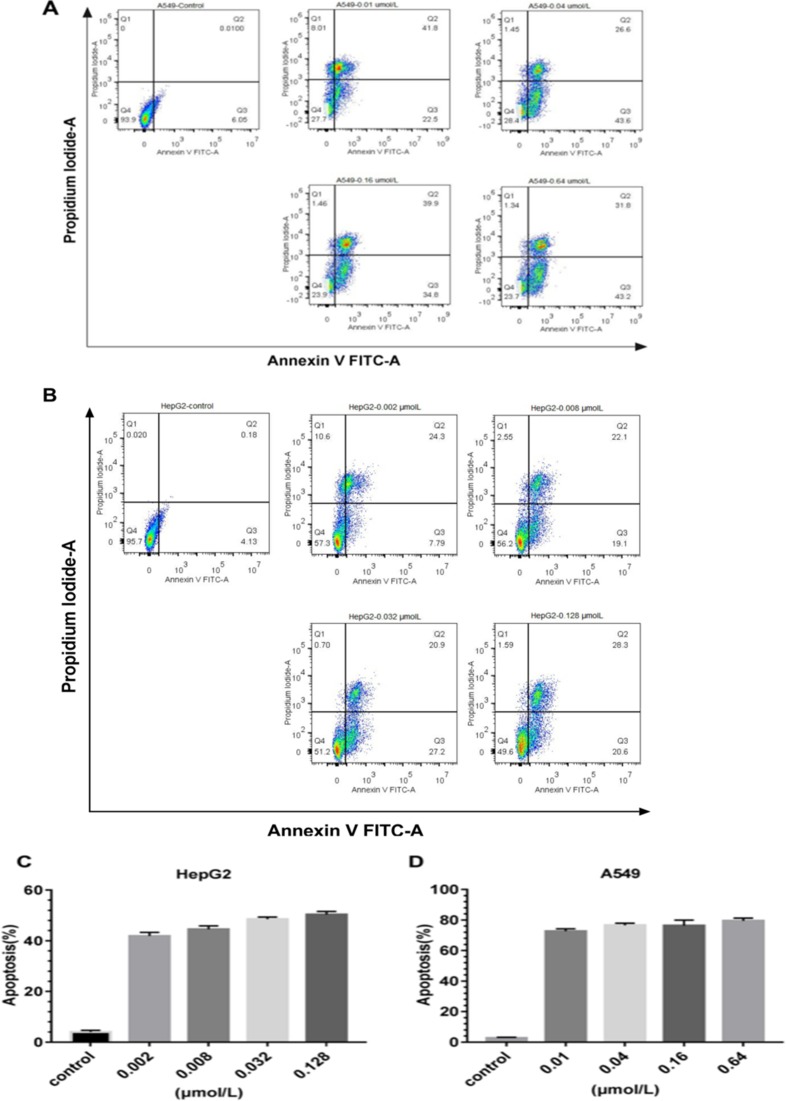
Apoptosis analysis of the immunotoxins rE/CUS (**A**) on A549, which incubated with 0.01, 0.04, 0.16 and 0.64μmol/L of rE/CUS for 72 h, dot blots and the percentage of the four quadrants. (**B**) on HepG2, which was incubated with 0.002, 0.008, 0.032, and 0.128 μmol/L of rE/CUS for 72 h, dot blots and the percentage of the four quadrants. (**C** and **D**) Early and late apoptosis or necrosis was combined in a column diagram foreach cell line for A549 and HepG2.

The proliferation of EGFR highly expression cells was evidently inhibited by rE/CUS. We then investigated whether the cellular growth inhibitor was induced via apoptosis. We examined HepG2 and A549 cells by using Annexin V-FITC/PI. After the cells were treated with gradient concentrations of rE/CUS for 72 h, the significant apoptosis of it induced by rE/CUS can be observed (Figure 7). The lower right quadrant indicates the proportion of cells in early apoptosis, the upper right quadrant shows the population of the late apoptotic or necrotic cells, and the upper left quadrant shows the necrotic cells. The total percentage of apoptosis includes early and late stages of apoptotic cells. Cell apoptosis were significantly induced by different concentrations of rE/CUS. For HepG2 cell line, approximately 40% of the cells were killed in corresponding concentration, and the apoptosis of A549 was around 70%. The untreated group showed a weak or even no apoptotic effect.

## DISCUSSION

EGFR overexpression is correlated with the increased aggressiveness, metastases, and poor prognosis in various cancers [39], including head and neck cancer, colorectal cancer, pancreatic, lung cancer, renal cell, prostate carcinoma and malignant glioma [40–41]. Therefore, it is necessary for anti-EGFR therapeutic strategies to be improved.

Monoclonal antibodies (mAbs) is a crucial part of the development of cancer therapy [42–43]. One of it's anti-tumor mechanisms is through binding the receptors that are expressed on the cellular surface or secreted in body fluids, after that the signal transduction pathways of cancer cells would be influenced, resulting the inhibition of tumor's proliferation and angiogenesis. Also, because of the presence of the intact Fc domain, the mAbs have the potential to induce antibody-dependent cell-mediated cytotoxicity (ADCC) by recruiting effector immunocytes and cytokines of immune system into the tumor microenvironment, which tend to further enhance the antitumor efficacy. Further more, as monoclonal antibodies can be used as a payload of non-specific cytotoxin, forming into nanomedicines (e.g. auristatin, maytansine, or doxorubicin) [39], it is capable of causing tumor specific cytotoxic effects [44]. Recently, the first antibody-drug conjugates(ADCs), Trastuzumab emtansine, was approved in treating patients with HER2-positive locally advanced breast cancer which is unresectable [45–46], indicating a bright future of the utilization of ADCs in clinic .

Target EGFR therapeutic monoclonal antibodies, namely cetuximab, panitumumab and some small tyrosine kinase inhibitors (TKIs), have gotten approval in treating colorectal cancer [47]. Other therapeutic monoclonal antibodies including gefitinib, lapatinib and erlotinib have been approved to be clinically effective in cancer treatment [48–51]. Nevertheless, the primary or acquired resistance of TKIs limits the application of these drugs, which are likely to be related with the constitutive stimulation of downstream molecules or over-expression of other tyrosine-kinase receptors [52]. For example, the sustaining activation of the downstream signaling paths MAPK and PI3K/Akt tend to promote cell proliferation, survival, differentiation and motility [53]. And angiogenesis causing up-regulated expression of the vascular endothelial growth factor (VEGF) on human cancer cells by it's ligands such as EGF and TGF-α can also be a factor to induce resistance to EGFR blocking agents [54]. Unlike TKIs, the immunotoxins rE/CUS, like D2C7-(scdsFv)-PE38KDEL which can be activated in glioblastoma patients expressing wild-type EGFR only or co-expressing wild-type EGFR and EGFRvIII [55], is solely depends on the expression of EGFR rather than tyrosine kinase signaling cascade triggered by EGFR. The target moiety of rE/CUS specifically binds to EGFR overexpression tumor cells and internalized the new type I RIP mediated by receptor, then the adenosine from the 28S ribosomal RNA were removed from RIPs, as a result,the RNA translation was interfered, leading to the protein biosynthesis inhibition [56–57].

Nanobody 7D12, with 15 KDa molecular mass sterically blocks it's ligand by binding to EGFR in a cetuximab-like manner [40]. Compared with traditional antibody, 7D12 alleviates immunogenicity result from particle size, and may shorten circulating time in the blood [58]. Previous research has shown that CUS is capable of inhibiting the proliferation of various tumor cells significantly, with a lethal activity four to seven times stronger than that of other type I RIPs, namely trichosanth, luffaculin, and amaranth protein [59].

To some extent, the advantages of recombinant immunotoxins outweigh chemically-linked conjugates. As integrate molecules, recombinant immunotoxins can be easily produced and purified [60–61]. Their cytotoxic potency, stability and affinity can be modified through genetic engineering technology [62–63]. In the mean time, the heterogeneity of conjugation products and dose-limiting side effects such as vascular leak syndrome can be mitigated by humanization or reconstruction [64].

This study aimed to construct a new EGFR-specific recombinant immunotoxin rE/CUS, and evaluate it's antitumor activity *in vitro*. The recombinant immunotoxin rE/CUS was constructed by connecting the 7D12 gene to the CUS with a flexible linker (G_4_S) _3_, The expression vector pET23 (a) containing desired gene was then transferred into prokaryotic expression system, and the Ni^+^ affinity chromatography column were used in the process of rE/CUS purification. Western blot analysis exhibiting the mouse anti-CUS and mouse anti-HIS specific band, proving that the protein is a soluble. The mass of protein rE/CUS is approximately 42 KDa by theoretical calculation, and the SDS-PAGE analysis of the purified protein showed that the bands were what we expected. These results demonstrate that we constructed a new recombinant immunotoxin.

The cytotoxic activity of rE/CUS was assessed for it's anticancer capability. Compared with 7D12 alone, the recombinant immunotoxins rE/CUS could effectively stick to EGFR highly expressing cells, which means that the antigen binding capability of rE/CUS was not affected by the extra-components of CUS and the delivery of CUS producing an impressive cytotoxic effects on tumor cell lines. rE/CUS significantly inhibited the cell viability against various EGFR highly expression tumor cell lines in a dose-and time-dependent manner. The cytotoxic activity of rE/CUS against cell lines HepG2, A549 and SW116 which the EGFR expression were up-regulated were highly sensitive to the rE/CUS. Whereas those whose EGFR expression were lower (SW620) showed no cell-lethal effects. The IC50 values of rE/CUS in different cell lines were much lower than those of CUS, and the IC50 values of 7D12 alone showed no difference with control group. The same dose of rE/CUS has different effects in different times tend to result from the internalization of the toxin [65]. Moreover, rE/CUS also significantly reduced the proliferation of HepG2 and A549 by inducing > 70% and > 40% apoptosis at a series of concentration. The specific toxicity of rE/CUS on HepG2 and SW116 were much stronger than other cell lines, with the IC50 value at 2.15 nmol/L and 2.33 nmol/L.

The binding ability of rE/CUS was lower than cetuximab and trastuzumab in the same concentration (data not shown). Chemically-linked conjugates Cetuximab-Cucurmosin and Trastuzumab- Cucurmosin were also constructed by our group [66–67]. Cetuximab-Cucurmosin were added in cultural medium of human colorectal cancer cell lines HCT116, SW480 for 5 day, and normal liver cells Lovo was used as negtive control cells. The IC50 of it was (0.075 ± 0.02) pmol/L, (0.058 ± 0.012)pmol/L, (0.511 ± 0.063)pmol/L which showed a significant time and dose-dependent proliferation inhibition, and the tumor inhibition rate of Cetuximab-Cucurmosin (20μg) were 48.625%. The IC50 of Trastuzumab-Cucurmosin on human breast cancer cell lines BT474 and human ovary cancer cell lines SK-OV-3 for 5 day were (0.0227 ± 0.007)nmol/L, (0.00252 ± 0.0054)nmol/L. The IC50 of Trastuzumab-Cucurmosin on BT474 is lowerer than Chemically-linked conjugates trastuzumab-DM1 (T-DM1) and Trastuzumab-deBouganin conjugates(T-deB) [68]. It is believed that the CUS is a promising toxin molecule for cancer therapy. In order to improve the binding ability of rE/CUS, we further designed and constructed an immunotoxin based CUS using a bivalent or biparatopic nanobodies as the target moiety. rGel-based immunotoxins used in cancer therapy showed that there is a strong correlation between the internalization percentage via receptor-mediated endocytosis [69]. Bivalent or biparatopic nanobodies have more intensive ability to block EGFR activation than monovalent nanobodies do. Previous studies have also demonstrated that bispecifc single-chain immunotoxins, such as CONAN-1 which elicits a potent inhibitory effect on tumour growth [39], were more effective than monospecifc or bivalent immunotoxins *in vitro* and *in vivo* [70–71]. Meanwhile, as nanobodies can be easily formatted that it can be constructed into bispecifc immunotoxins, like bivalent immunotoxin PG002, which engage two different epitopes or antigens on cancer cells [72].

In conclusion, a new recombinant immunotoxin linking 7D12 with CUS was designed, constructed, and expressed. It is specifically lethal to EGFR highly expression tumor cell lines *in vitro*. Our results exhibited that rE/CUS could be a potential therapeutic strategy in treating EGFR-positive solid tumors.

## MATERIALS AND METHODS

### Materials

pET-32a vector (+) and *E.coli* BL21 (DE3), his-tagged fusion protein kit and western blot antibodies were obtained from Sangon Biotch in Shanghai. The Plasmid Midi Kits were purchased from Gene Mark, and mRNA isolation kits were from CWBIO.

Human hepatoma cell HepG2, human NSCLC A549, human colorectal cancer cell SW116 and human colon cancer cell SW620 cell lines were acquired from cell bank / stem cell bank of the Chinese Academy of Sciences. RPMI-1640 supplemented with 0.292 g/L L-glutamine, 2g/L sodium hydrogen carbonate, and 800 U/L gentamicin were used for cells culture. Humidified incubator with temperature of 37°C, CO_2_ concentration of 5%, were used for cells maintaining.

### Methods

### Construction of aprokaryotic-expressing plasmid

Competitive inhibitor 7D12 was generated with Ablynx NV as described previously [12]. The gene- encoding CUS was obtained from our laboratory patent(CN 101215327A) whichwere already constructed in plasmid. 7D12 and CUS sequence was amplified through PCR from plasmids pET-32a-7D12 and pET-32a-CUS, the 7D12 gene was then connected to CUS with the linker (G_4_S) _3_ by overlapping PCR. After agarose gel electrophoresis was performed, the amplified products were digested, purified, ligated into plasmid vector pET-32a with Ndel and Xhol restriction enzyme cutting sites linking with the target fragment and vector. The fusion gene rE/CUS was cloned into *E.coli* expression vector, pET-32a (+). The expression vector pET-32a (+) -rE/CUS was identified though restriction enzyme digestion and DNA sequencing.

### Protein expression, isolation, and purification

The expression vector pET-32a (+)-rE/CUS was transfected into BL21 (DE3) E.coli cells. The bacterial culture medium containing 50μg/ml ampicillin were shaken at 200rpm at 37°C until the it reached the log phase where A600nm is approximately 0.6. Then 200 μg/ml Isopropyl β-D-1-thiogalactopyranoside (IPTG) were added for protein expression induction. Before the bacterial cultures were harvested through centrifugation (8000 rpm, 20 min, 4°C), the bacterial culture medium were shaken at 200 rpm at 25°C for 16 h. Then the pellets were resuspended in 0.015mol PBS after 8000rpm washing for 10 min at 4°C. As a total volume of 3L bacterial culture centrifuged into pellet then resuspended in 30 ml Tris-NaCl and 30 mg lysozyme, and pulse-sonicated for 90 min on ice, the supernatant of solution was seperated at 8000 rpm for 10 min 4°C. In purification process, the specimen was washed with 50 mmol Tris, 300 mmol NaCl, 15 mmol imidazole, and the protein was eluted with 50 mmol Tris, 300 mmol NaCl, and 150 mmol imidazole according to His-tagged fusion protein kit. Then it was examined through sodium dodecyl sulfate polyacrylamide gel electrophoresis (SDS-PAGE). Finally, the product expressed was dialyzed, filter-sterilized, and stored at 4°C.

### Western blot analysis

Purified protein was analyzed through Western blot analysis with a mouse anti-HIS antibody and mouse anti-CUS antibody as primary antibody. The horseradish peroxidase-(HRP-) labeled goat anti-mouse IgG was selected as the secondary antibody.

### Flow cytometry of EGFR expression on different cell lines

Human hepatoma cell HepG2, human non-small cell lung cancer A549, human colorectal cancer cell SW116 and human colon cancer cell SW620 was detected by FACS Calibur (BD Biosciences) at Alexa flour 488. Scatchard plots were generated and analyszed using FlowJo V10. The cells were harvested at 2000 rpm for 5 min, then washed at 2000 rpm, for 5 min by PBS containing 1% bovine serum albumin (BSA). The cetuximab was used as the primary antibody and the anti-human-FITC was used as the second antibody at 4°C for 30 min. The cells were washed and analyzed by flow cytometry. Each experiment was repeated thrice.

### Flow cytometric detection of rE/CUS binding capability

rE/CUS binding capability was detected by FACS Calibur (BD Biosciences). HepG2, A549, SW116 and SW620 cells were harvested, 2000 rpm, 5min, then washed with PBS containing 1% BSA, 2000 rpm, and 5min. then cells were incubated with the 30 μM rE/CUS for 30min at 4°C. The cells were incubated with mouse anti-HIS antibody for 30min at 4°C, and then incubated with mouse anti-HIS-APC for 30 min at 4°C. The cells were washed and analyzed using flow cytometry. Each results was testified thrice.

### *In* vitro cytotoxicity assays

### Detecting of Inhibition of rE/CUS Proliferation for 72 h

Positive cells (HepG2, A549 and SW116) overly express EGFR, and negative cells (SW620 cell) were seeded in 96-well plates with 4 × 10^4^/ml per well, then the cells were incubated for 24 h to adherence in 5% CO_2_ at 37°C. When in the logarithmic phase, the cells were subsequently treated with rE/CUS, CUS and 7D12 at different concentrations and incubated for 72 h at 5% CO_2_, 37°C. Before detection, trichloroacetic acid was added, and the cells were incubated at 4°C overnight. And washed five times with H_2_O. Sulforhodamine B (SRB). The cells were incubated for 30 min at room temperature, washed five times with 1% acetic acid, and 100nmol tris-(hydroxymethyl)- amino-methane was added for absorption testing (515 nm) [73]. Each experiment was repeated thrice.

### Dose-and time-dependence of the inhibitory effect of rE/CUS on the proliferation of HepG2, and A549 cells

HepG2 and A549cells were seeded in 96-well plates with 3 × 10^4^/ml per well when cells in logarithmic phase, then the cells were incubated for 24 h at 5%CO_2_, 37°C. Subsequently, cells were treated with rE/CUS, 7D12 and CUS at different concentrations, and incubated for 2–5 days at 5% CO_2_, 37°C. Then the cell proliferation in each plate was detected at the corresponding time by SRB.Each experiment was repeated thrice.

### Flow cytometric analysis of cells apoptosis

AnnexinV-FITC/propidium iodide (PI) detection kit (KeyGEN BioTech) was used to analyze cells apoptosis induced by rE/CUS. A549 and HepG2 were seeded in 6 wells plates with 4 × 10^4^/ml, and incubated for 24 h at 5% CO_2_, 37°C, later on A549 and HepG2 were treated with rE/CUS for 72 h. Then the cells were harvested, washed twice with PBS containing 2% BSA, and resuspended in binding buffer at 1 × 10^5^ cells/ml. 5 μL AnnexinV-FITC and 5μL PI were added for 15 min at room temperature in the dark for staining. The cells were washed and resuspended in 500 ul binding buffer, and flow cytometry were applied for analyzing. Each results was testified thrice.

### Statistical analysis

SPSS 19.0 were used for analyzing the means ± standard deviation (SD) of results, and image processing was using GraphPad Prism 7.00. Signifcant difference of 2 groups (*P* < 0.05)were compared by Student's *t*-test.
